# Feasibility and safety of an F16 small-caliber catheter-based, irrigation-free protocol in BPH patients undergoing green laser enucleation of the prostate: a single-center retrospective cohort study

**DOI:** 10.3389/fmed.2026.1961968

**Published:** 2026-10-01

**Authors:** Xin Gao, Kaibo Guo, Yijun Fu, Beixun Xu, Puzhao Liang, Jinfeng Xu, Jie Pan, Yongtong Ruan

**Affiliations:** 1 Department of Urology, Yangjiang Hospital of Traditional Chinese Medicine, Affiliated to Guangzhou University of Chinese Medicine, Yangjiang, Guangdong, China; 2 Department of Urology, Yangjiang Hospital of Traditional Chinese Medicine, Yangjiang, Guangdong, China

**Keywords:** benign prostatic hyperplasia (BPH), F16 catheter, GreenLight laser, non-irrigation protocol, perioperative outcomes

## Abstract

**Objective:**

GreenLight laser enucleation of the prostate (GreenLEP) offers superior hemostasis, raising the possibility of omitting postoperative continuous bladder irrigation. This study aimed to evaluate the feasibility and safety of an F16 small-diameter catheter non-irrigation protocol in BPH patients.

**Methods:**

A retrospective single-center study included 300 consecutive BPH patients undergoing GreenLEP at Yangjiang Hospital of Traditional Chinese Medicine between January 2024 and June 2025. The exposure was implementation of the F16 non-irrigation protocol. The primary outcome was successful completion without continuous bladder irrigation or secondary hemostatic intervention. Covariates included age, prostate volume (PV), operative time, ASA score, anticoagulant/antiplatelet use, urinary retention, preoperative catheterization, and concomitant bladder stones. Stratified analysis and ROC curve analysis were performed to explore predictors of failure.

**Results:**

Overall, 265 patients (88.3%) successfully completed the non-irrigation protocol. Median hemoglobin decrease was 2.0 g/L (IQR 1–3), with no blood transfusions. In the parsimonious multivariable logistic regression model adjusted for age, prostate volume, ASA classification, and anticoagulant/antiplatelet use, prostate volume was not independently associated with MajorFailure (adjusted OR 1.14 per 10-mL increase, 95% CI 0.76–1.71, *P* = 0.527). ROC analysis suggested a PV cutoff of 76.35 mL for predicting failure (AUC 0.65, 95% CI 0.55–0.75).

**Conclusion:**

The F16 non-irrigation protocol after GreenLEP is safe and feasible, with high success rates and minimal perioperative bleeding. Increased PV and anticoagulant therapy modestly reduce success but do not preclude application. These findings support broader implementation of non-irrigation strategies to enhance postoperative comfort and recovery.

## 1 Introduction

Benign prostatic hyperplasia (BPH) is among the most prevalent urological conditions affecting middle-aged and elderly men, with incidence rising sharply with advancing age (1). Data from the Global Burden of Disease (GBD) study indicate that the number of prevalent BPH cases among men aged ≥60 years reached approximately 79 million in 2019—a more than 120% increase compared with 1990—and this upward trajectory is projected to continue through 2035 (2). Meta-analytical estimates place the global prevalence of BPH at approximately 26.2%, with roughly 45% of men over 45 years of age and up to 80% of those over 70 affected (3). In China, a pooled epidemiological analysis reported a BPH prevalence of approximately 36.6% among men aged ≥40 years (4), and, driven by accelerating population aging, China has become one of the countries bearing the greatest global BPH disease burden. Disease progression may result in bladder outlet obstruction (BOO) with potentially severe sequelae, including acute urinary retention, recurrent hematuria, bladder calculi, recurrent urinary tract infections, and upper urinary tract dilatation with consequent renal insufficiency—all of which substantially impair health-related quality of life and impose considerable burden on healthcare systems (5). Transurethral surgery of the prostate remains the primary surgical intervention for BPH, with procedural volumes continuing to rise globally year on year.

In recent years, technological advances in laser surgery have brought growing attention to green laser enucleation of the prostate (GreenLEP), which offers favorable hemostatic properties, a low perioperative hemorrhagic risk, and broad applicability to large-volume prostates (6). Conventional postoperative management following transurethral prostate surgery typically involves placement of a large-caliber (≥F20) three-way urethral catheter with continuous bladder irrigation (CBI) to prevent clot formation and catheter obstruction (7). However, CBI is associated with clinically significant limitations. The mechanical stimulation generated by large-bore catheters and continuous irrigation frequently causes catheter-related bladder discomfort (CRBD), with a reported postoperative incidence of 47%–90% (8). Bladder spasm occurs in 11%–28% of cases, typically manifesting as persistent, cramping perineal pain that substantially compromises patient comfort and early recovery (9). Furthermore, CBI imposes a considerable nursing burden, restricts patient mobility, and prolongs hospitalization—all of which are incompatible with the core principles of Enhanced Recovery After Surgery (ERAS) (10). The GreenLight laser (532 nm) operates at a wavelength that coincides precisely with the dual absorption peaks of oxyhemoglobin, conferring highly selective absorption by hemoglobin with minimal uptake by water (11). This optical property enables superior tissue vaporization and hemostatic coagulation, generating a coagulation layer approximately 1–2 mm in depth on the prostatic fossa surface (12). As a result, accurate enucleation is achieved while bleeding vessels are effectively sealed, yielding intraoperative and postoperative hemorrhagic risks substantially lower than those associated with conventional transurethral resection (13). These hemostatic characteristics provide a biological rationale for minimizing the need for postoperative irrigation. In this context, an F16 catheter was selected as the initial postoperative drainage catheter to balance adequate urinary drainage with minimization of catheter-related irritation. Current adult catheterization guidance supports the use of the smallest catheter caliber consistent with effective drainage. Evidence directly addressing catheter size after GreenLEP remains limited, and peri-catheter urinary leakage was not systematically recorded in our cohort; therefore, the present study cannot directly quantify the risk of leakage associated with F16 catheterization.

Preliminary evidence supporting an irrigation-free approach following laser prostatectomy has begun to emerge. A study from China demonstrated that, in appropriately selected patients undergoing holmium laser enucleation of the prostate (HoLEP), an irrigation-free protocol was non-inferior to CBI with respect to early postoperative complications, catheterization duration, length of hospital stay, and functional outcomes at 1–6 months of follow-up, establishing both the feasibility and safety of this approach (14). Nevertheless, the existing literature is subject to several important limitations: (1) most published data derive from small case series or single-arm comparisons, limiting methodological rigor; (2) the evidence base is predominantly focused on HoLEP, while dedicated investigations of irrigation-free strategies following GreenLEP remain exceptionally scarce; and (3) no study to date has systematically quantified the risk factors associated with irrigation-free protocol failure, leaving clinicians without validated patient selection criteria. Consequently, the safety boundaries and predictors of failure for an F16 catheter–based, irrigation-free management strategy after GreenLEP have yet to be adequately characterized.

To address these gaps, we conducted a single-center retrospective cohort study enrolling BPH patients who underwent GreenLEP with postoperative F16 catheter placement and omission of CBI at Yangjiang Hospital of Traditional Chinese Medicine between January 2024 and June 2025. Patient demographic characteristics, perioperative parameters, and complication profiles were systematically collected to evaluate the feasibility and safety of this management strategy. Stratified analyses (by prostate volume, operative duration, and anticoagulant use) and receiver operating characteristic (ROC) curve analysis were performed to identify key determinants of irrigation-free protocol success and to establish optimal prostate volume cutoff values. The distinguishing feature of this study lies in its focus on the real-world feasibility of an F16 small-bore catheter, irrigation-free protocol, rather than conventional risk factor profiling alone. Our findings are expected to provide evidence-based guidance for optimizing perioperative BPH management, supporting the reduction of unnecessary CBI use, shortening catheterization and hospitalization duration, enhancing patient comfort, and enriching the literature on enhanced recovery following laser prostatectomy.

## 2 Methods

### 2.1 Study design and patient selection

This retrospective single-center study included consecutive patients who underwent Green laser enucleation of the prostate (GreenLEP) for benign prostatic hyperplasia (BPH) at Yangjiang Hospital of Traditional Chinese Medicine between January 2024 and June 2025. All procedures were performed by experienced surgeons using a standardized surgical technique.

Patients managed postoperatively with an F16 urethral catheter without routine continuous bladder irrigation (CBI) were eligible for inclusion. Patients managed postoperatively with an F16 urethral catheter as part of the initial non-irrigation pathway were eligible for inclusion. Patients who were placed on routine continuous bladder irrigation as their initial postoperative management, without an initial trial of the F16 non-irrigation protocol, were excluded. Patients who initially entered the F16 non-irrigation pathway but subsequently required manual irrigation or continuous bladder irrigation because of clinically indicated postoperative bleeding or clot-related events were retained in the cohort and classified as protocol failures.

### 2.2 Surgical procedure and postoperative management

GreenLEP was performed under general or spinal anesthesia using a standard endoscopic enucleation technique. Hemostasis was carefully achieved intraoperatively using the Green laser system. At the completion of surgery, an F16 urethral catheter was routinely inserted.

Unlike conventional postoperative management protocols, at the completion of surgery, an F16 urethral catheter was routinely inserted, and patients initially entered the non-irrigation pathway without routine continuous bladder irrigation. Patients were closely monitored for urine color, catheter drainage, clot retention, and postoperative bleeding. If clinically significant bleeding or clot-related obstruction occurred, manual irrigation or continuous bladder irrigation was initiated as rescue management. Patients requiring such escalation after initially entering the non-irrigation pathway were classified as protocol failures. The catheter size was selected to provide adequate postoperative urinary drainage while minimizing catheter-related urethral and bladder irritation. In accordance with current urological catheterization guidance, a 16-Fr catheter is an appropriate size for adults with light hematuria or small clots when continuous irrigation is not routinely required, whereas larger-bore catheters are generally reserved for more substantial hematuria, clot burden, or the need for bladder irrigation.

Patients with concomitant bladder stones underwent simultaneous endoscopic lithotripsy during the same procedure.

### 2.3 Data collection

Baseline demographic and perioperative data were retrospectively collected from electronic medical records. Variables included age, preoperative hemoglobin level, prostate volume (PV), ASA score, hypertension, diabetes mellitus, anticoagulant or antiplatelet medication use, urinary retention, preoperative catheterization status, and concomitant bladder stones.

Perioperative variables included operative time, postoperative hemoglobin decrease (*Δ*Hb), length of hospital stay, clot retention, manual irrigation, continuous bladder irrigation, cystoscopic clot evacuation, blood transfusion, secondary hemostatic intervention, and readmission within 30 days.

### 2.4 Definition of study outcomes

The primary outcome of this study was the feasibility of the F16 non-irrigation protocol.

Successful protocol completion was defined as postoperative management without the need for continuous bladder irrigation or secondary invasive intervention.

MajorFailure was defined as the occurrence of any predefined postoperative bleeding-related escalation requiring intensification of management, including continuous bladder irrigation, cystoscopic clot evacuation, secondary hemostatic intervention, or other clinically significant postoperative bleeding-related events requiring escalation of care. Because some escalation events could occur sequentially in the same patient, individual complication categories were not mutually exclusive. Patients whose clinically significant postoperative bleeding-related events did not involve the specific interventions listed above were classified as “other clinically significant postoperative bleeding-related events.”

### 2.5 Stratified feasibility analysis

Exploratory stratified analyses were performed to examine the feasibility of the F16 non-irrigation protocol across clinically relevant procedural and bleeding-related characteristics. Prostate volume, operative time, and anticoagulant/antiplatelet use were selected because they represent distinct clinical dimensions potentially relevant to non-irrigation management: adenoma burden, procedural burden, and bleeding tendency, respectively. These analyses were intended to explore clinically meaningful patterns rather than to provide an exhaustive assessment of all baseline characteristics.

Patients were stratified into three prostate volume groups (<60 mL, 60–100 mL, and >100 mL) and three operative time groups (<60 min, 60–90 min, and >90 min).

Receiver operating characteristic (ROC) curve analysis was performed specifically for prostate volume because it is a continuous preoperative measure directly reflecting prostate/adenoma burden and was significantly associated with MajorFailure in the univariable analysis. The ROC analysis was intended to explore whether prostate volume could provide a clinically interpretable threshold for protocol failure; it was not intended to establish a comprehensive multivariable prediction model.

### 2.6 Statistical analysis

Continuous variables were expressed as mean ± standard deviation (SD) or median with interquartile range (Q1–Q3), as appropriate, and categorical variables were presented as frequencies and percentages. Between-group comparisons were performed using Student's t-test, Mann–Whitney U test, Pearson's chi-square test, or Fisher's exact test, as appropriate.

Univariable logistic regression analysis was used to examine the associations between clinically relevant baseline characteristics and MajorFailure. A parsimonious multivariable logistic regression model was subsequently constructed to assess whether prostate volume was independently associated with MajorFailure after adjustment for potential confounding factors. Age, prostate volume, ASA classification, and anticoagulant/antiplatelet use were included in the multivariable model based on their clinical relevance and the need to limit model complexity given the 35 MajorFailure events. Prostate volume was modeled as a continuous variable and reported per 10-mL increase. Odds ratios (ORs), 95% confidence intervals (CIs), and two-sided *P* values were calculated.

Exploratory stratified analyses were performed according to prostate volume, operative time, and anticoagulant/antiplatelet use. Receiver operating characteristic (ROC) analysis was performed to assess the discriminative performance of prostate volume for MajorFailure. The area under the ROC curve (AUC) with 95% CI, optimal cutoff value, sensitivity, specificity, positive predictive value (PPV), negative predictive value (NPV), and Youden's index were calculated. The ROC-derived cutoff was considered exploratory and was not interpreted as a validated standalone clinical decision threshold.

All statistical analyses were performed using R software version 4.3.2 and Jamovi version 2.4.11. A two-sided *P* value <0.05 was considered statistically significant.

## 3 Results

### 3.1 Patient demographics and baseline characteristics

A total of 300 patients were included in the study, among whom 265 (88.3%) successfully completed the F16 non-irrigation protocol and 35 (11.7%) experienced protocol failure. Baseline characteristics of both groups are presented in Table 1. Compared with the success group, patients in the failure group were older (72.6 ± 6.6 vs. 68.9 ± 7.0 years; t = −3.01, *P* = 0.003) and had lower preoperative hemoglobin levels (124.1 ± 12.5 vs. 131.3 ± 12.8 g/L; t = 3.13, *P* = 0.002). The median prostate volume was significantly larger in the failure group [81.4 mL [IQR: 60.1–108.7] vs. 66.5 mL [IQR: 46.9–85.0]; Z = −2.96, *P* = 0.003].

**Table 1:** Baseline characteristics of patients stratified by F16 irrigation-free protocol outcome.

Variables	Total (*n* = 300)	Success (*n* = 265)	Failure (*n* = 35)	Statistic	*P* value
Age (years), mean ± SD	69.32 ± 7.03	68.88 ± 6.98	72.63 ± 6.55	t = −3.01	0.003
Preoperative Hb (g/L), mean ± SD	130.44 ± 12.97	131.28 ± 12.83	124.09 ± 12.45	t = 3.13	0.002
Prostate volume (mL), median (Q1, Q3)	67.55 (47.88, 86.65)	66.50 (46.90, 85.00)	81.40 (60.10, 108.70)	Z = −2.96	0.003
ASA classification, *n* (%)				*χ*^2^ = 10.58	0.005
Grade 1	81 (27.0)	76 (28.7)	5 (14.3)		
Grade 2	140 (46.7)	127 (47.9)	13 (37.1)		
Grade 3	79 (26.3)	62 (23.4)	17 (48.6)		
Hypertension, *n* (%)	185 (61.7)	156 (58.9)	29 (82.9)	χ^2^ = 7.53	0.006
Diabetes mellitus, *n* (%)	62 (20.7)	54 (20.4)	8 (22.9)	χ^2^ = 0.12	0.733
Anticoagulant/antiplatelet use, *n* (%)	55 (18.3)	43 (16.2)	12 (34.3)	χ^2^ = 6.73	0.009
Urinary retention, *n* (%)	82 (27.3)	65 (24.5)	17 (48.6)	χ^2^ = 9.00	0.003
Preoperative catheterization, *n* (%)	104 (34.7)	85 (32.1)	19 (54.3)	χ^2^ = 6.73	0.009
Concomitant bladder calculi, *n* (%)	34 (11.3)	23 (8.7)	11 (31.4)	χ^2^=13.74	<0.001

Hb, hemoglobin; SD, standard deviation; Q1, first quartile; Q3, third quartile; ASA, American Society of Anesthesiologists physical status classification; t, independent samples t-test; Z, Mann–Whitney U test; χ^2^, Pearson's chi-square test.

Higher ASA scores were observed among patients who experienced failure (ASA grade 3: 48.6% vs. 23.4%; *χ*^2^ = 10.58, *P* = 0.005). Comorbid conditions such as hypertension (82.9% vs. 58.9%; *χ*^2^ = 7.53, *P* = 0.006), use of anticoagulant/antiplatelet drugs (34.3% vs. 16.2%; *χ*^2^ = 6.73, *P* = 0.009), presence of urinary retention (48.6% vs. 24.5%; *χ*^2^ = 9.00, *P* = 0.003), preoperative catheterization (54.3% vs. 32.1%; *χ*^2^ = 6.73, *P* = 0.009), and coexisting bladder calculi (31.4% vs. 8.7%; *χ*^2^ = 13.74, *P* < 0.001) were more frequent in the failure group (Table 1). No significant difference was observed in the prevalence of diabetes mellitus between groups (22.9% vs. 20.4%; *χ*^2^ = 0.12, *P* = 0.733).

These findings indicate that patients who experienced protocol failure tended to have advanced age, larger prostate volume, higher ASA scores, and several comorbid conditions, suggesting that baseline patient characteristics are associated with protocol outcomes.

### 3.2 Perioperative outcomes and postoperative complications

A total of 300 patients underwent the F16 non-irrigation protocol following GreenLEP. Overall, 265 patients (88.3%) successfully completed the protocol, whereas 35 patients (11.7%) experienced major failure requiring additional intervention. Perioperative outcomes are summarized in Table 2. The median operative time was 83.0 min (Q1–Q3: 70.0–98.3), and the median postoperative hemoglobin decrease (*Δ*Hb) was 2.0 g/L (Q1–Q3: 1.0–3.0), indicating effective intraoperative hemostasis.

**Table 2:** Perioperative outcomes and postoperative complications stratified by protocol outcome.

Variable	Overall (*n* = 300)	Success (*n* = 265)	Failure (*n* = 35)
Protocol outcome			
Successful non-irrigation protocol, *n* (%)	265 (88.3)	265 (100.0)	0 (0.0)
Protocol failure, *n* (%)	35 (11.7)	0 (0.0)	35 (100.0)
Intraoperative parameters			
Operative time (min), median (Q1–Q3)	83.0 (70.0–98.3)	83.0 (69.0–97.0)	93.0 (75.5–116.5)
*Δ*Hb (g/L), median (Q1–Q3)	2.0 (1.0–3.0)	2.0 (1.0–3.0)	3.0 (2.0–3.0)
Postoperative recovery			
Length of hospital stay (days), median (Q1–Q3)	3.0 (3.0–4.0)	3.0 (2.0–4.0)	5.0 (3.0–6.0)
Postoperative complications/escalation events			
Clot retention, *n* (%)	24 (8.0)	0 (0.0)	24 (68.6)
Manual irrigation, *n* (%)	15 (5.0)	0 (0.0)	15 (42.9)
Continuous bladder irrigation, *n* (%)	11 (3.7)	0 (0.0)	11 (31.4)
Cystoscopic clot evacuation, *n* (%)	3 (1.0)	0 (0.0)	3 (8.6)
Other clinically significant postoperative bleeding-related events, *n* (%)	11 (3.7)	0 (0.0)	11 (31.4)
Blood transfusion, *n* (%)	0 (0)	0 (0)	0 (0)
Secondary hemostatic intervention, *n* (%)	1 (0.3)	0 (0.0)	1 (2.9)
Readmission within 30 days, *n* (%)	0 (0)	0 (0)	0 (0)

Data are presented as *n* (%) unless otherwise indicated. Postoperative complication and escalation events were not mutually exclusive; therefore, their frequencies should not be summed. Continuous bladder irrigation, cystoscopic clot evacuation, and secondary hemostatic intervention were components of the MajorFailure definition. Eleven patients were classified as MajorFailure because of other clinically significant postoperative bleeding-related events not represented by the specific intervention categories above.

The median length of hospital stay was 3.0 days (Q1–Q3: 3.0–4.0), reflecting rapid postoperative recovery. Postoperative complications and bleeding-related escalation events are summarized in Table 2. All recorded clot-related and bleeding-related intervention events occurred among patients classified as protocol failures. Clot retention occurred in 24 patients (8.0%), all of whom were in the failure group. Manual irrigation was required in 15 patients (5.0%), continuous bladder irrigation in 11 patients (3.7%), cystoscopic clot evacuation in 3 patients (1.0%), and secondary hemostatic intervention in 1 patient (0.3%); each of these events occurred in the failure group. In addition, 11 patients (3.7%) were classified as MajorFailure because of other clinically significant postoperative bleeding-related events that did not involve the specific interventions listed above. These postoperative events were not mutually exclusive and therefore should not be summed. No patients required blood transfusion, and no 30-day readmissions were recorded.

### 3.3 Stratified feasibility analysis of the F16 non-irrigation protocol

Exploratory stratified analyses were performed according to prostate volume, operative time, and anticoagulant/antiplatelet use (Table 3). Success rates decreased with increasing prostate volume (PV): 92.0% (104/113) in patients with PV <60 mL, 88.7% (126/142) in those with PV 60–100 mL, and 77.8% (35/45) in those with PV >100 mL (*P* = 0.041). These findings indicate that a substantial proportion of patients with prostate volumes >100 mL completed the protocol successfully, although the failure rate was higher than in patients with smaller prostates. These findings indicate that larger prostate volume was associated with a higher rate of protocol failure, although 77.8% of patients with PV >100 mL still successfully completed the non-irrigation protocol. Thus, larger prostate volume may reduce the likelihood of successful irrigation-free management but does not preclude protocol completion in most patients with large prostates.

**Table 3:** Stratified feasibility analysis of the F16 non-irrigation protocol.

Variable	Success (*n* = 265)	Failure (*n* = 35)	*P* value
Prostate volume (mL)			0.041
<60	104 (92.0)	9 (8.0)	
60–100	126 (88.7)	16 (11.3)	
>100	35 (77.8)	10 (22.2)	
Operative time (min)			0.108
<60	41 (93.2)	3 (6.8)	
60–90	128 (90.1)	13 (9.9)	
>90	96 (83.5)	19 (16.5)	
Anticoagulant/antiplatelet use			0.018
No	222 (90.7)	23 (9.3)	
Yes	43 (78.2)	12 (21.8)	

Data are presented as *n* (%). PV: prostate volume. *P* values were derived from Pearson's chi-square test.

When stratified by operative time, success rates were 93.2% (<60 min), 90.1% (60–90 min), and 83.5% (>90 min), respectively (*P* = 0.108). Although a trend toward increased failure was observed with longer operative duration, the difference did not reach statistical significance, indicating that the protocol remains feasible across a range of operative durations.

With respect to anticoagulant or antiplatelet therapy, the success rate was 90.7% (222/245) among patients not receiving such medications, compared with 78.2% (43/55) among those receiving them (*P* = 0.018) (Table 3). Although anticoagulant or antiplatelet use was associated with a lower protocol completion rate, the majority of patients in this subgroup still completed the protocol successfully. This finding indicates that protocol completion was possible in some patients receiving anticoagulant or antiplatelet therapy, supporting the potential feasibility of the strategy in appropriately selected patients receiving antithrombotic therapy.

### 3.4 Multivariable analysis of factors associated with MajorFailure

Among the 300 patients, 35 (11.7%) experienced MajorFailure. In univariable logistic regression analysis, older age, larger prostate volume, anticoagulant/antiplatelet use, and higher ASA classification were associated with MajorFailure (Table 4).

**Table 4:** Univariable and multivariable logistic regression analysis of factors associated with MajorFailure.

Variable	Univariable OR	95% CI	*P* value	Adjusted OR	95% CI	*P* value
Age, per 1-year increase	1.08	1.03–1.14	0.004	1.02	0.87–1.19	0.817
Prostate volume, per 10-mL increase	1.26	1.09–1.44	0.001	1.14	0.76–1.71	0.527
Anticoagulant/antiplatelet use	2.69	1.25–5.82	0.012	1.26	0.40–3.90	0.693
ASA grade 2 vs. grade 1	1.56	0.53–4.54	0.418	0.89	0.17–4.72	0.894
ASA grade 3 vs. grade 1	4.17	1.46–11.93	0.008	1.17	0.07–18.37	0.912

OR, odds ratio; CI, confidence interval; ASA, American Society of Anesthesiologists.

Prostate volume was analyzed as a continuous variable and ORs are expressed per 10-mL increase. ASA grade 1 was used as the reference category. Adjusted ORs were obtained from the parsimonious multivariable logistic regression model including age, prostate volume, ASA classification, and anticoagulant/antiplatelet use.

In the parsimonious multivariable logistic regression model adjusted for age, prostate volume, ASA classification, and anticoagulant/antiplatelet use, prostate volume was not independently associated with MajorFailure. For each 10-mL increase in prostate volume, the adjusted odds ratio was 1.14 (95% CI 0.76–1.71, *P* = 0.527). Age (adjusted OR 1.02, 95% CI 0.87–1.19, *P* = 0.817), anticoagulant/antiplatelet use (adjusted OR 1.26, 95% CI 0.40–3.90, *P* = 0.693), and ASA classification (ASA grade 2 vs. grade 1: adjusted OR 0.89, 95% CI 0.17–4.72, *P* = 0.894; ASA grade 3 vs. grade 1: adjusted OR 1.17, 95% CI 0.07–18.37, *P* = 0.912) were also not independently associated with MajorFailure.

### 3.5 ROC analysis

ROC analysis demonstrated modest discriminative performance of prostate volume for MajorFailure (Figure 1), with an AUC of 0.654 (95% CI 0.553–0.754). The exploratory optimal cutoff was 76.35 mL, with a sensitivity of 62.9%, specificity of 66.0%, positive predictive value of 19.6%, negative predictive value of 93.1%, and Youden's index of 0.289. These findings indicate that prostate volume alone has limited ability to discriminate patients who will experience MajorFailure, and the 76.35-mL threshold should not be considered a standalone preoperative risk-stratification criterion.

**Figure 1:**
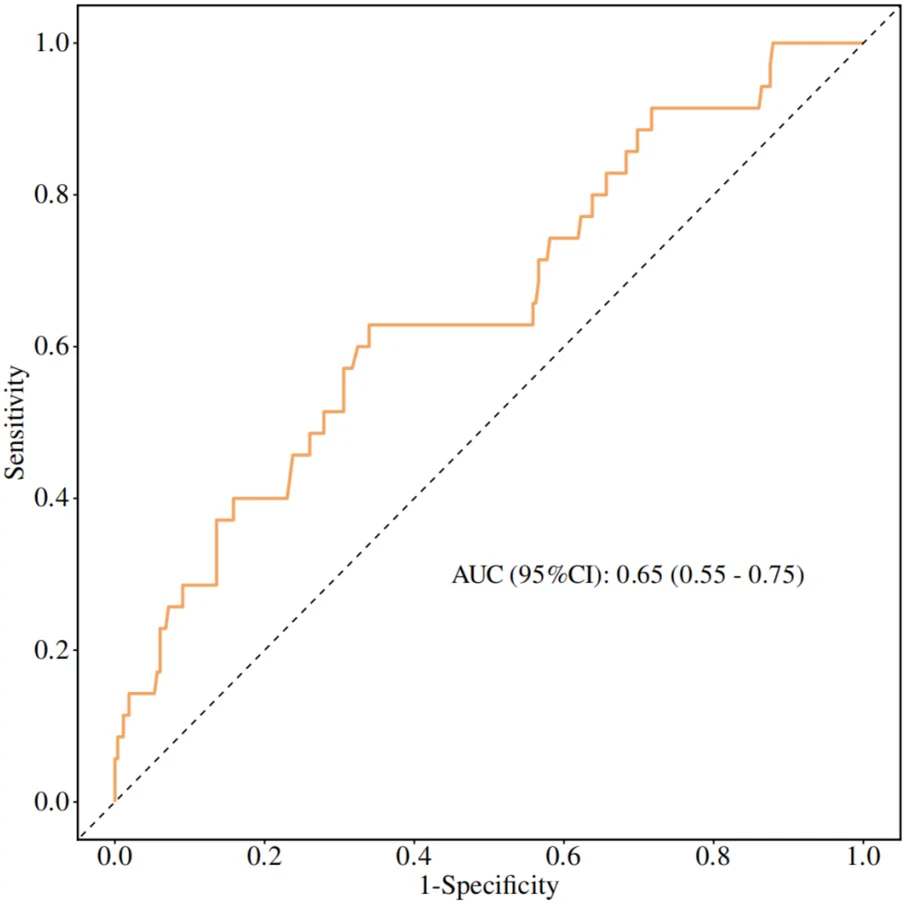
Receiver operating characteristic (ROC) curve of prostate volume for predicting MajorFailure of the F16 non-irrigation protocol after GreenLEP.

The ROC curve showed modest discriminative performance, with an AUC of 0.654 (95% CI 0.553–0.754). The exploratory optimal cutoff was 76.35 mL.

## 4 Discussion

In this single-center retrospective cohort study of 300 consecutive BPH patients undergoing GreenLEP, we evaluated the feasibility of an F16 small-caliber catheter-based, irrigation-free postoperative protocol. Overall, 265 patients (88.3%) successfully completed the protocol without requiring continuous bladder irrigation or secondary invasive intervention, whereas 35 patients (11.7%) met the criteria for MajorFailure. Clot retention occurred in 24 patients (8.0%), while continuous bladder irrigation, cystoscopic clot evacuation, and secondary hemostatic intervention were required in 11 (3.7%), 3 (1.0%), and 1 (0.3%) patients, respectively. These findings indicate a high protocol completion rate within the selected cohort; however, because all patients underwent GreenLEP and there was no conventional CBI comparator group, the observed postoperative events cannot be causally attributed to the surgical procedure or the non-irrigation strategy alone.

The present findings are broadly consistent with emerging evidence supporting reduced or omitted postoperative bladder irrigation after endoscopic prostate enucleation; however, evidence from HoLEP should not be directly extrapolated to GreenLEP because the procedures differ in laser-tissue interaction and tissue handling. GreenLEP uses a 532-nm laser preferentially absorbed by hemoglobin, producing photothermal tissue vaporization and coagulation, whereas HoLEP uses a 2140-nm pulsed holmium laser strongly absorbed by water, enabling precise tissue incision with a limited coagulation zone; these differences, together with variations in enucleation and tissue-handling techniques, may influence intraoperative hemostasis, residual postoperative oozing, tissue debris, and clot accumulation (15, 16). Evidence for postoperative non-irrigation is currently more established for HoLEP: Hao et al. compared 62 patients managed without bladder irrigation with 150 receiving continuous bladder irrigation after HoLEP, and after propensity score matching of 47 pairs, found no significant difference in early postoperative complications, supporting omission of routine irrigation in appropriately selected patients (14); nevertheless, these findings do not establish an equivalent clot-related risk profile after GreenLEP. Comparative studies have reported broadly comparable perioperative safety outcomes between GreenLEP and HoLEP, including a 2025 propensity score-matched analysis, although that study did not specifically evaluate postoperative non-irrigation and reported some differences in longer-term functional outcomes and late complications (17). Thus, current evidence supports the general feasibility of both enucleation approaches but does not establish equivalence between GreenLEP and HoLEP regarding clot formation or postoperative irrigation requirements. The present study therefore provides GreenLEP-specific evidence for an F16 non-irrigation pathway, while the occurrence of clot retention and subsequent rescue irrigation in a subset of patients demonstrates that effective laser hemostasis does not eliminate postoperative clot-related events. Accordingly, omitting routine irrigation should be considered a strategy for selected patients rather than a universal requirement-free approach after GreenLEP, and prospective comparative studies are needed to determine whether differences in laser-tissue interaction and tissue handling translate into clinically meaningful differences in postoperative bleeding, clot formation, and irrigation requirements.

The higher failure rate observed in our cohort may instead reflect the specific demands of postoperative non-irrigation management. A larger adenoma generally requires removal of a greater tissue volume and may result in a larger postoperative prostatic fossa with a greater exposed vascular surface, potentially increasing the likelihood of residual postoperative oozing. Under conventional CBI, continuous bladder washout may reduce the accumulation of blood and small clots. When irrigation is omitted, even modest persistent oozing may be more likely to result in clot accumulation and catheter obstruction, thereby triggering rescue irrigation and classification as protocol failure. Thus, prostate volume may be more relevant to the feasibility of an irrigation-free postoperative pathway than to the overall technical safety of GreenLEP itself. Importantly, 77.8% of patients with PV >100 mL successfully completed the protocol in our cohort, indicating that large prostate volume should be considered a factor associated with reduced protocol success rather than an absolute contraindication to irrigation-free management. This interpretation remains exploratory and should be validated in larger prospective cohorts.

The feasibility of the F16 non-irrigation protocol following GreenLEP may be partly explained by the hemostatic properties of the GreenLight laser. The 532 nm wavelength is selectively absorbed by oxyhemoglobin, concentrating photothermal energy within red blood cells to achieve near-instantaneous vascular sealing and tissue vaporization, while simultaneously generating a 1–2 mm subepithelial coagulation layer that occludes microvessels and acts as a thermal barrier against delayed hemorrhage (17, 18). This mechanism may reduce bleeding from the prostatic fossa and thereby reduce the likelihood of clinically significant clot formation, although postoperative clot-related events may still occur and may depend on the extent of residual oozing and tissue handling (19, 20). Clinical evidence further supports this physiological rationale. In a contemporary series of 180-W GreenLight laser prostatectomy performed in patients with uncorrectable bleeding tendency, Elshal et al. demonstrated low perioperative bleeding morbidity, with blood transfusion required in only 4.3% of high-risk patients and none in the lower-risk group, confirming the robust hemostatic profile of GreenLight-based surgery even in compromised coagulation settings (21). In addition, long-term data on GreenLight photoselective vaporization have shown extremely low transfusion rates (approximately 0.4%), which has been attributed to the selective hemoglobin absorption and superior coagulative properties of the 532-nm laser (13). Taken together, the synergistic optical and thermal characteristics of the GreenLight laser—selective hemoglobin absorption, efficient vaporization, and microvascular coagulation—create a hemostatic environment conducive to catheter-only postoperative management, thereby providing both the mechanistic foundation and clinical justification for integrating the F16 non-irrigation protocol into an ERAS pathway for laser prostate enucleation.

Several limitations should be considered. First, the retrospective, single-center, single-arm design introduces the potential for selection and information bias and, importantly, the absence of a conventional CBI comparator precludes causal separation of procedure-related adverse events from those related to the irrigation-free postoperative strategy. The observed complication rates should therefore be interpreted as outcomes associated with the combined GreenLEP and F16 non-irrigation pathway rather than as estimates of the independent effect of either component. Second, the relatively small number of MajorFailure events (*n* = 35) limits the statistical power of multivariable analyses and meaningful subgroup comparisons. Third, the study focused on perioperative outcomes, and long-term functional recovery and health-related quality of life were not assessed. Finally, the single-center setting limits external validation and generalizability. Prospective comparative studies of GreenLEP with and without routine postoperative irrigation are needed to determine comparative safety, identify protocol-specific risks, and establish the generalizability of these findings.

In conclusion, the F16 non-irrigation protocol following GreenLEP showed a high protocol completion rate in this selected single-center retrospective cohort. The findings support the feasibility of this postoperative management strategy in appropriately selected patients but do not establish comparative safety or a causal effect of non-irrigation on postoperative complications. Prospective comparative studies are needed to validate these findings and determine the appropriate patient selection for irrigation-free postoperative management.

The ROC curve showed modest discriminative performance, with an AUC of 0.654 (95% CI 0.553–0.754). The exploratory optimal cutoff was 76.35 mL.

## Data Availability

The data analyzed in this study is subject to the following licenses/restrictions: The data supporting the findings of this study are available from the corresponding author upon reasonable request. Requests to access these datasets should be directed to Xin Gao, 2424609849@qq.com.
